# Innovative mental health policies, plans and interventions: How to manage consequences of economic crisis?

**DOI:** 10.1192/j.eurpsy.2021.215

**Published:** 2021-08-13

**Authors:** J.M. Caldas De Almeida

**Affiliations:** Lisbon Institute Of Global Mental Health, Nova Medical School, Nova University of Lisbon, Lisboa, Portugal

## Abstract

**Abstract Body:**

Available evidence shows that countries may shield their population’s exposure and vulnerability to mental health risks during and after an economic recession by strengthening their policies and reorienting their budgets. Populations’ mental health protection during economic crises can only be achieved by the policies of different sectors. Social protection, social programmes and social safety nets proved to be fundamental buffers against inequalities in mental health. Several actions have proven to be effective in this area, including measures to improve social protection, reduce income inequalities, and mitigate the impacts of unemployment. To address the negative consequences of unemployment, active labour market programmes, including special programmes for unemployed young people and families, programmes to promote the employment of people with disabilities, and debt relief programmes should be implemented. The response of the health system is critical. During and after economic recessions, it is fundamental to ensure the responsiveness and effectiveness of the mental health system. To attain this goal, mental health services that are closer to the populations and that facilitate the early identification of mental health problems and the implementation of integrated interventions should be strengthened. The latter is a crucial approach to tackle the mental health problems that more often worsen in periods of economic instability, such as depression, suicidal behaviour and heavy drinking. A special attention should also be dedicated to strengthening the network of community-based mental health services, promoting the integration of mental health in primary care, and enhancing the coordination between mental health services and social care.

**Disclosure:**

No significant relationships.

